# A Scoping Review to Identify Barriers and Enabling Factors for Nurse–Patient Discussions on Sexuality and Sexual Health

**DOI:** 10.3390/nursrep11020025

**Published:** 2021-04-16

**Authors:** Maria Åling, Agnes Lindgren, Hillevi Löfall, Leah Okenwa-Emegwa

**Affiliations:** 1 Department of Health Sciences, The Swedish Red Cross University College, P.O. Box 1059, 141 21 Huddinge, Sweden; alim@rkh.se (M.Å.); agneslindgren93@gmail.com (A.L.); hillevi.lofall@hotmail.com (H.L.); 2 Department of Public Health and Sport Science, Faculty of Health and Occupational Studies, University of Gävle, 801 76 Gävle, Sweden

**Keywords:** nurse, sexuality, sexual health, barriers, enabling factor, power, well-being, norm, care values, ethics

## Abstract

Background: Sexuality and sexual health (SSH) are essential aspects of care that have evolved since a 1975 World Health Organization (WHO) report on SSH. However, nurses still consider discussing the subject with patients a challenge. This scoping review aimed to map, synthesize, and summarize findings from existing literature regarding barriers and enabling factors for nurse–patient SSH discussions in care contexts. Methods: A scoping review model inspired by Arksey and O’Malley was used to search for and synthesize studies published between 2009 and 2019. The databases searched were the Cumulative Index to Nursing and Allied Health Literature (CINAHL) and Medical Literature Analysis and Retrieval System Online, i.e., MEDLARS Online. A total of nineteen articles were eligible to be included. Results: Two main categories of enabling factors were identified, i.e., a professional approach via using core care values and availability of resources. Three major categories of barriers were identified: beliefs and attitudes related to age, gender, and sexual identity; fear and individual convictions; and work-related factors. Conclusions: Applying professionalism and core care values as well as making resources available are likely to promote SSH discussions between nurses and patients. Moreover, there is a need for a norm-critical approach in education and practice.

## 1. Introduction

Addressing sexuality and sexual health (SSH) is an essential aspect of healthcare that has evolved over the years; however, health professionals, including nurses, still consider it a difficult subject [1,2]. Sexuality is integral to human beings throughout their lifetime [3], therefore, sexual health should be ensured by having a positive and respectful approach to sexuality and sexual relationships [3,4]. From a care perspective, sexual wellbeing can be categorized into three domains, i.e., sexual wellbeing integral to holistic care, sexual wellbeing associated with other health conditions, and sexual wellbeing related to specific sexual problems and infections [5]. Therefore, the role of nurses in counseling and discussions on SSH cannot be overemphasized. However, although one of the fundamentals of health counseling is shared power and control to create an interactive relationship [6], socially defined roles in healthcare contexts may prevent meaningful nurse–patient discussions. Socially defined healthcare roles often portray the healthcare professionals as custodians of health knowledge and givers of information; thus, patients are expected to rely on health professionals for when and how information is delivered and its content [6,7,8]. It will be interesting to understand the implications of the foregoing for SSH discussions.

According to Holmgren (2017), due to increasing diversity in patient and nursing staff demographics, nurses must think globally even when providing nursing care locally [9]. They should reflect on their engagement within complex societies to counteract social injustices [9]. However, perceptions about SSH are influenced by a complex array of factors such as social, economic, political, cultural, legal, historical, and religious [3]. They may have implications for the nurse–patient SSH discussions. For example, although sexuality is a lifelong component [3], there are misconceptions about sexual activity and age, e.g., that sex decreases with aging, which is an assumption contradicted in research [10]. Contextual differences such as upbringing, social learning [11], media exposure, and the environment [12] also seem to foster age and gender-related differences regarding attitudes to sexual relationships. The influence of religion and culture on prevailing norms and attitudes regarding sexuality is well documented [13] and indicates that heteronormativity is common. Even in countries that rank high in terms of human rights and gender equality, the Lesbians, Gay, Bi-sexual, Trans-sexual, or Queer (LGBTQ) community report being more exposed to violence, discrimination, and lack of acceptance in specific social contexts than the rest of the population [14,15]. The resulting fear, lack of freedom of expression among LGBTQ persons, and other factors common to the general population may have implications for SSH discussions.

Discussing SSH in healthcare is relevant for good sexual wellbeing, recovery, general health, and patients’ desire for information [5,16,17]. Thus, the role of SSH discussions in healthcare contexts for preventing inequalities in sexual health and wellbeing cannot be overemphasized [13]. More than forty years after The World Health Organization’s (WHO) report on SSH in healthcare [1], and despite various research studies highlighting the significance of sexual health, many nurses across the globe still consider SSH a difficult subject. The consequence is the creation of inequities and inequalities in sexual health [18], i.e., differences in measurable health outcomes in individuals and across population groups [2,19,20]. It is considered unjust to allow preventable and unnecessary health differences to persist [21]. Such systematic differences, which are avoidable if reasonable means are used, are known as health inequity [20,22]. Health inequities have significant social and economic costs to individuals and society [20]. According to the WHO, failure to provide adequate SSH-related information and services to any individual or population group is a violation of human rights contributing to inequality in sexual health [18]. Unfortunately, not many nurses are willing to ask their patients about SSH [23,24,25]. Furthermore, insufficient SSH content remains a major challenge across nursing programs, although this may vary across geographical locations due to social–cultural factors [5,25].

While there are research findings highlighting barriers to incorporating SSH discussions in healthcare, there is also emerging evidence regarding enabling factors for SSH discussions. Therefore, using a scoping approach, it is possible to summarize vital evidence [26]. Scoping review is a form of research synthesis used for mapping available literature on a particular topic or research area to identify key concepts, gaps in the research, and types and sources of evidence that can inform practice, policymaking, and research [27]. Thus, knowledge gained from mapping barriers and enabling factors for discussing SSH can potentially provide a possible framework for addressing the subject in education and practice.

### AIM

This scoping review aims to summarize barriers and enabling factors for the nurse–patient SSH discussions within healthcare.

## 2. Materials and Methods

The Preferred Reporting Items for Systematic Reviews and Meta-Analyses (PRISMA) checklist was used for this study. More specifically, the PRSIMA checklist for scoping reviews [28] was used to identify barriers and enabling factors for discussing SSH in healthcare and to structure the presentation of the findings. Scoping reviews help to summarize vital evidence on a topic without necessarily going through the process of a formal systematic review [26]. Knowledge production from scoping reviews often forms a part of a knowledge-to-action cycle, and the information generated is applied in practice, policy development, and research [26,27]. This study’s scoping review model is inspired by Arksey and O’Malley (2005) and involves six stages: identifying the research question; searching for relevant studies; study selection; charting the data; collating, summarizing, and reporting the results; and consulting with stakeholders.

Only studies published between 2009 and 2019, which investigated SSH discussions between nurses and patients, were eligible for inclusion. Due to the interest to get a broader picture, eligibility was not limited to any geographical area. The exclusion criteria applied were based on the study population, i.e., all studies focusing on only students were excluded. To capture a wider range of studies, studies with qualitative and quantitative designs were included, while literature reviews were excluded. A search was conducted in two databases to find relevant articles i.e., Cumulative Index to Nursing and Allied Health Literature (CINAHL) and Medical Literature Analysis and Retrieval System Online, also known as MEDLARS Online.

The search was conducted between January and February 2019 using relevant search terms such as Nurs*, Sexual health, Sexual*, Sexuality, “Attitude to Sexuality”, “Communication”, and “Communication Barriers”. Truncation (i.e., *) was used in some instances to include multiple suffixes in the search result. An initial broad search using, for example, “Nurs*” “health”, “sexual health” was conducted. Boolean operators “AND” and “OR” were used to narrow down or broaden the search respectively and as deemed necessary. For example, search combinations were Nurs* AND “discussing sexuality”, Nurs* AND “Sexual topics”, OR “Patient’s sexual health”. Table 1 and Table 2 show the search matrix and the eligible articles from each search, respectively. The Mesh thesaurus was useful for determining appropriate search terms [29]. Although CINAHL headings are derived from Mesh terms (i.e., Medical Subject headings), they contain more healthcare-related terms [30]. Subject headings in both databases may slightly differ from each other; however, the search terms applied for this study were the same in both databases and included sexual health, sexuality, attitude to sexuality, communication, and communication barriers.

### 2.1. Consulting with Stakeholders

Findings from this scoping review were presented at a seminar. Participants at the seminar were nurses and public health scientists, most of whom were researchers and lecturers in diverse nursing fields, including sexual health. Feedback from the seminar was used to refine and restructure findings.

### 2.2. Ethical Consideration

No ethical permission was needed as there was no direct contact with human subjects. Eligible articles contain statements on ethical aspects where relevant. Data were treated on an aggregate level, thereby reducing the possibility of identifying individual participants.

## 3. Results

**Figure 1:**
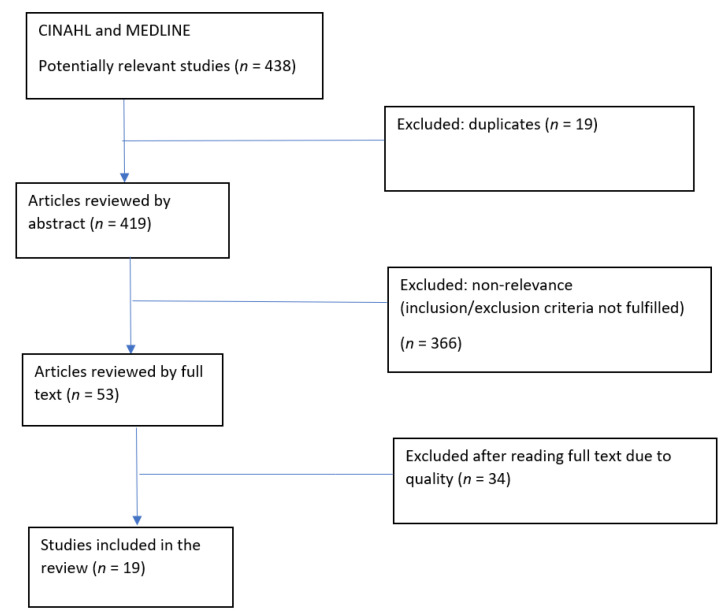
Search process flow chart.

A total of 438 articles were identified; nineteen out of these were duplicates, and a further 366 were removed due to non-relevance. Quality assessment was done using a checklist for literature reviews from the Swedish Agency for Health Technology Assessment and Assessment of Social Services [31]. The assessment was via scoring individual articles on parameters such as level of systematic errors, transferability, and precision, among others. Only nineteen articles met the cut-off for medium and high quality and are included in this review (see Figure 1). Relevant information extracted from the articles includes objectives, study population, location of the study, research question, methods, and results, i.e., barriers and enabling factors. Data were synthesized, mapped, and interpreted to identify barriers and enabling factors related to healthcare professionals–patient SSH discussions.

Table 3 shows a total of nineteen articles included in the review (i.e., eleven quantitative and eight qualitative studies). Many of the survey instruments used in quantitative studies covered areas such as participants’ demographics, SSH training, comfort levels discussing SSH with patients and attitudes. For studies with a qualitative design, the interview guides included open-ended questions on whether SSH is discussed, experiences, perceptions, barriers, and likely solutions, among others. The articles were from the Netherlands, Sweden, UK, Turkey, USA, Canada, Australia, New Zealand, Thailand, China, and Zimbabwe (see Table 2). Findings revealed enabling factors and barriers for discussing SSH. The two major categories of enabling factors were professional approach, including core care values, and availability of resources. Three main categories of barriers identified were beliefs and attitudes related to age, gender, and sexual identity; fear and individual convictions; and work-related factors.

### 3.1. Enabling Factors

#### 3.1.1. Professional Approach, including Core Care Values

Nurses who discussed SSH with their patients viewed this task as part of their professional responsibility to alleviate patients’ and family members’ suffering [32]. They tend to use various strategies to approach the subject; for example, they look for appropriate moments, e.g., in a private room [32] and when discussing physical problems or treatment [33,34,35,36]. Moreover, most nurses believe it feels more professional to talk about SSH in terms of sexual function and “mechanically” functioning of the body rather than, for example, from a relationship perspective [33]. By using humor and ice breakers (especially with male patients), nurses and patients felt more relaxed to discuss SSH [37]. Nurses felt appreciated by patients and satisfied that the patients appeared relieved following such discussions [32].

Trust and good care-relation were two other factors that were considered most useful for discussing SSH [32,33,36,38]. For example, Zeng et al. (2012) found that most nurses believed good care-relation and good communication skills were keys to handling problems related to patients’ sexual health [38]. However, while an established care relationship between nurse and patient facilitates SSH discussion, a long care-relation may have the opposite effect [33].

#### 3.1.2. Availability of Resources

Several studies showed that education and training are enabling factors [32,35,39,40,41,42,43,44,45,46]. Hoekstra et al. (2012) suggest that various workshops based on identified barriers to discussing SSH are needed to expand nurses’ perspectives [35]. Examples of training with a specific focus include practical training in communication skills for nurses [39], SSH-specific training [43], and capacity building for discussing SSH with LGBTQ patients [40]. Furthermore, clear routines, policies, discussion guides, and checklists were useful for smooth discussions [35,37,40,44,45,46].

Nurses tend to discuss SSH if they have supportive colleagues to consult with (e.g., when they felt uncertain about the right answers to patient questions) and if inter-professional collaboration (e.g., referring patients to physiotherapists and sexologists) was possible [42]. Support from other professionals through regular reflections and guidance [40] was also an enabling factor. Findings show that although not all nurses who had worked longer were willing to discuss SSH with their patients [33], nurses older than 30 years and those with more than ten years working experience were more comfortable discussing SSH with patients [47]. This group of nurses generally served as useful support for younger nurses who turn to them when faced with difficult questions from patients (such as being sexual active while on a catheter).

### 3.2. Barriers for Discussions on Sexuality and Sexual Health

#### 3.2.1. Beliefs and Attitudes Related to Age, Gender, and Sexual Identity

Nurses avoided asking older patients about their sexuality and sexual health due to perceived difficulty taking up the subject with this group [33,34,35,36,44,46,48,49]. Moreover, there is a preconceived notion that patients in their eighties (80s) were not sexually active [36]. Nurses often considered older patients as asexual or uninterested in sex [46]. According to Van Ek et al. (2017), staff working with dialysis patients did not raise the issue as part of routine interaction, especially with older patients [44]. Although nurses agreed that asking older patients about SSH was important, seven out of ten nurses avoided discussing SSH with patients older than 76 years, mostly because it reminded them about their elderly parents or grandparents [33]. Discussing SSH with younger patients was easier due to the openness among patients in this category and the opportunity to assume a “parental” role [36].

The gender of the nurse and the patient was an important factor. While female nurses found it more challenging to discuss the subject with their male patients, male nurses reported similar difficulty in discussing with their female patients [34,46,48]. In the study by Akinci (2011), nurses expressed discomfort with asking and answering male patients about SSH, examining reproductive organs, and giving advice and information to patients with erectile problems [48].

Initiating SSH discussions with LGBTQ patients was considered difficult and more challenging than with heterosexual patients [34,46,48]. Half of the nurses in the study by Martels et al. (2017) expressed a desire for more training and knowledge on discussing SSH and other matters related to LGBT/HBTQ [40].

#### 3.2.2. Fear and Individual Convictions

Nurses avoided raising SSH with patients due to fear of making patients uncomfortable or making them feel insulted [33]. In one study, although close to 70% of nurses reported having adequate knowledge about the impact of diseases and treatments on sexual health, up to about 40% of themchose not to raise the subject because they considered it embarrassing [50] and that some patients considered SSH a subject too private to discuss with nurses [32,34,39]. Nurses’ fear, worry, and personal convictions about the necessity of SSH discussion may also depend on the individual patient’s peculiar circumstances. An example is in psychiatric care, where nurses fear that asking patients intimate questions may lead to misinterpretation, upsetting the patient, or worsening their condition [41]. Another example is the belief that SSH discussions may not be necessary for palliative care patients [46].

Several studies showed that the inability to lay aside personal convictions is a barrier to SSH discussions and had implications for whether nurses chose to broach the subject [33,40,46]. For example, the conviction that the use of contraceptives by a 14-year-old is morally wrong prevented a nurse from offering contraceptives to adolescents [40]. Another example of personal convictions prevailing over professional requirements is the tendency to classify some patients as too sick to care about SSH during hospital admission [34,39,49,50].

Although ethnic differences may cause communication barriers, nurses’ and patients’ ethnicity and cultural background and religion play important roles in how comfortable or uncomfortable nurses felt when taking up the subject [32,33,35,40,44,45,46,48,49]. Maree and Fitch (2019) found that nurses hardly asked about SSH to avoid misunderstanding or “stepping on patients’ toes” [49]. Furthermore, nurses in Canada reported that patients often initiated the conversation; in contrast, nurses in Zimbabwe expressed that patients did not initiate such conversation due to fear that nurses may eventually disclose the discussions’ contents to other people [49].

#### 3.2.3. Work-Related Factors

Although most nurses acknowledge their responsibility to have SSH discussions [35,36,43,46], uncertainty regarding role descriptions plays a role in whether SSH discussion will be initiated. Whereas some nurses considered it the patient’s responsibility to initiate discussions, others claimed discussing SSH is doctors’ responsibility [33,39,40,44,46,47,49]. Added to unclear roles is lack of time, which is a common recurring factor preventing nurses from discussing SSH [34,36,40,46,49]. Thus, discussing SSH is less prioritized, especially when nurses had too many patients under their care at a given period [45] or during staff shortage [39].

Many nurses reported a lack of knowledge regarding SSH discussion techniques [33,34,36,39,41,42,43,44,45,46,47]. Although insufficient undergraduate education content is often cited as the cause [36,45], inadequate knowledge is also work-related. Two examples were identified; the first is a lack of care context-specific training (e.g., patients with urine catheter [33], radiotherapy patients [47]). The second is nurses’ uncertainty regarding what information to give to patients if a problem related to sexual health is raised by patients or identified by the nurse [36,41]. Some nurses try to address their lack of knowledge by asking colleagues for advice, reading professional publications, researching the internet, or using personal experiences or the experiences of people they know [32]. However, nurses report feeling uncertain about the quality of the information they have acquired independently.

Many of the studies reveal a lack of routine regarding how and when SSH discussion should be initiated. For example, nurses would avoid the subject if they did not find an appropriate opportunity or if a third party was in the room [44]. Other work-related barriers include a lack of screening tools, a lack of checklists and conversation guides for discussing SSH [40,46], and a lack of knowledge regarding existing models for evaluating sexual health [45]. Evcili and Demirel (2018) found that up to 86% of nurses were not aware of models for evaluating sexual health [45].

## 4. Discussion

Our findings show that despite the diverse study contexts of the studies included in this scoping review and their peculiarity (e.g., cancer care, renal care, cardiology, primary care, psychiatry, geographical differences etc.), the barriers and enabling factors for SSH discussions were similar. Findings reveal enabling factors that can counteract common barriers to discussing SSH in healthcare settings.

### 4.1. Applying Professionalism through Core Care Values and Relevant Resources

Interestingly, factors that may logically be associated with an increased tendency to discuss SSH (e.g., awareness of SSH due to years of working experience) did not necessarily foster a tendency to discuss SSH with patients. On the other hand, nurses who view SSH in terms of the core values of caring, e.g., to alleviate the suffering experienced by patients and their families [51], were among the ones who discussed SSH [32]. This group did their best to overcome limitations, e.g., finding appropriate opportunities and create the right moments for bringing up SSH. Furthermore, barriers created by factors such as fear of upsetting the patient, of being misinterpreted, the perception that nurses lack time, among others, do not align with the fundamentals of health counseling. Health counseling is based on shared power and control to create an interactive relationship with the patient [6]. Our findings reveal that vital ingredients for SSH discussions and content were a professional approach based on core care values (such as trust and a good care-relationship) and addressing work-related issues (e.g., training, clear routines, guidelines, discussion templates, inter-professions collaborations, clarification of tasks/areas of responsibility, and allocation of time).

Trust, described as one of nursing’s intangible assets, is essential in relationship building and is closely associated with good care-relation [51,52]. Similarly, since patients are known to construct their power in interactions [53], it is likely that trust and good care-relation empower patients to construct their power positively. By doing so, the nurse—patient power asymmetry is reduced, and SSH discussions can hold without inhibitions. A professional approach to discussing SSH can also be enhanced by addressing work-related issues such as training, clear routines, guidelines, discussion templates, collaborations across professions, clarification of tasks/areas of responsibility, and time allocation. Using humor and other ice breakers made initiating the subject more relaxing for nurses and patients, especially male patients. Using this approach tactfully while maintaining a professional atmosphere is a successful strategy. More training in health counseling techniques via continuous education for practicing nurses and more SSH content in undergraduate education is vital. A professional approach to care prevents the exclusion of certain patient groups’ in SSH discussion and enhances patients’ satisfaction.

### 4.2. The Need for a Norm-Critical Approach in Nursing Education and Practice

Another critical aspect of ensuring an inclusive approach to SSH discussions in healthcare is through a norm-critical approach, as shown by findings from this scoping review. There is evidence that including norm awareness in nursing curricula can make learners more aware of their own perspectives and become equipped to challenge practices, structures, and routines that result in exclusion within healthcare [54,55]. Several factors identified from this scoping review (such as those related to age, gender, religion, ethnicity, and culture) are rooted in norms and beliefs that make SSH a taboo and sensitive topic for both patients and practitioners. Findings show that nurses in most studies avoided discussing SSH with older patients despite evidence that sexuality is a lifelong component that does not disappear with age [3,10]. Beliefs about SSH as a taboo subject may become heightened when it concerns the SSH of older patients [56]. In this review, most nurses either believe older patients are asexual or have difficulty discussing SSH with patients who remind them of their elder parents or grandparents. The reverse appears to be the case when dealing with younger patients; i.e., nurses tend to assume a parental role [36]. Similarly, SSH discussions’ contents may be subject to nurses’ personal convictions (often based on religious or cultural norms or both) about what is acceptable for various patient categories. Although the feelings of taboo around SSH were common in many of the studies, the impact of culture and religion in reinforcing the taboo around SSH was more pronounced in the studies from, for example, Turkey, Jordan, and Zimbabwe. Nursing practice is described as delivering quality unbiased care, advocacy, activism to counteract social injustices, and inequalities in health [9]. Globally, there is increasing diversity in patients’ and nurses’ demographics in terms of ethnicity, beliefs, host country language skills, and sexual identity. Therefore, nursing education and practice must adopt approaches that equip nurses to reflect on their engagement within complex societies and to think globally while working locally [9].

Norms and beliefs around gender were a barrier for discussing SSH (mostly if the patient was of the opposite gender) and a determinant for how patients’ SSH concerns are treated. For example, nurses avoided SSH discussions, especially with patients of the opposite gender, if they felt intrusive and overstepping boundaries [41]. Even when SSH is discussed, nurses tend to consider men’s sexual problems more concrete and more straightforward to address than those presented by women [36]. The consequence is that men get more empathy and treatment alternatives than women. Norms and beliefs around gender also influence SSH discussions with LGBTQ patients. Many healthcare systems are still heteronormative in their approach, for example, there is still limited terminology, and nurses lack knowledge about the SSH needs of LGBTQ patients [57]. Thus, these patients remain at the risk of discrimination and exclusion in SSH discussions [36].

Various reports and policy documents highlight the healthcare workforce’s crucial role in addressing inequalities and inequities in health [58,59]. These policies are anchored on core care principles and values such as trust, good care-relation, and others, which place the patients’ needs at the center. An example is patient-centered care, which is described as care consistent with patients’ values, needs, and desires and empowers patients to become active participants in their care [60,61]. Patient-centered care is characterized by communication, partnership, and health promotion [62]. Our findings suggest that applying core care values and having a norm-critical approach in practice can result in effective SSH communication by, for example, reducing the power imbalance between nurse and patient. The partnership created with patients makes it easier to find out their SSH needs. From a sexual health perspective, it is vital to determine patients’ sexual function and how planned medications, or surgery would, for example, affect their sexual health and their relationship with their partner [63]. Adopting some of the enabling factors identified in this review and adapting them to context-specific situations can potentially result in effective communication and partnership needed for sexual health promotion. Furthermore, identifying barriers and applying enabling factors for discussing SSH with patients can also reduce the uncertainties and anxieties faced by nurses regarding discussing SSH.

Some strengths and weaknesses of this study are worth mentioning. Unlike systematic reviews, scoping reviews generally do not assess the overall quality of evidence; however, they are useful for summarizing vital evidence [25]. While effort has been made to search broadly in two comprehensive databases for health research, it is still possible that relevant research (and therefore, their findings) have been missed out. Moreover, the review of only English language publications may also result in the possible exclusion of relevant research findings. These limitations notwithstanding, this study’s strength is the presentation of vital evidence relevant for practice and education.

## 5. Conclusions

The importance of discussing SSH within the healthcare setting cannot be overemphasized given its relevance for good sexual wellbeing, recovery, general health, and patients’ desire for information [5,16,17]. However, forty years since the World Health Organization’s (WHO’s) report on the importance of sexual health and the role of healthcare [1], the nurse–patient discussion remains a challenge. Although the global community of health professionals shares fundamental ethics and values upon which health professions are anchored, patient care is not free from the influence of prevailing cultural, social, economic, and religious norms [64]. People, whether nurse or patients, also take their beliefs with them wherever they go. While norms are good for meaning, identity, and society’s functioning, they also have the power to exclude [65]. Consequently, the absence of clear routines and guidelines for discussing SSH within the context of patient care may leave the initiation and content of such discussions entirely at the nurse’s discretion. Thus, inequalities in sexual health can result due to two possible reasons, i.e., the possible exclusion of specific patient categories and nurse–patient power asymmetry when SSH discussions are held [6,66]. Therefore, given the increasing diversity in patient and nursing population and that the principles of care include encountering everyone on their own terms, a norm-critical approach is vital in nursing education and practice [67].

### Implications for Practice

A norm-critical approach in education and practice will promote awareness and criticism of norms and power structures that result in exclusion within healthcare [54]. Adopting a professional approach (such as training, providing vital tools and resources, and applying core care values such as trust and good care relations) may foster SSH discussions. Thus, it is essential to adopt a more reflective approach both in practice and training so that nurses are challenged to question their individual assumptions and practices [51,68,69]. A reflective approach will be useful for norm awareness, fostering professionalism, and it will hopefully boost nurses’ readiness to initiate and discuss SSH without bias or inhibitions.

## Figures and Tables

**Table 1:** Search matrix.

Search	Date	Database	Search Terms	Number of Hits	Number of Abstracts Read	Full Text Read	No. of Eligible Articles
#1	28 January 2019	CINAHL complete Medline	Nurs* AND “discussing sexuality”	18	18	15	9
#2	28 January 2019	CINAHL complete Medline	Nurs* AND (“Talking to patients” OR Dialog* OR Address* OR Approach* OR Discuss* OR Communicat* OR “Sex talk”) AND (Sexual* OR “Sexual health”) AND (Barrier* OR Problem* OR Difficult* OR Challenge*)	315	314 (313) 1 duplicates	16 (15) 1duplicate	4 (3) 1 duplicate
#3	29 January 2019	CINAHL complete Medline	Nurs* AND (MH “sexual health”) OR (MH “Sexuality”) OR (MH “Attitude to Sexuality”) AND (MH “Communication”) OR (MH “Communication Barriers”)	64	64 (49) 15 duplicates	27 (12) 15 duplicates	12 (3) 9 duplicates
#4	30 January 2019	CINAHL complete Medline	Nurs* OR “Healthcare providers” AND Sex* AND “Talking to”	37	37	8	1
#5	4 February 2019	CINAHL complete Medline	Nurs* AND “Sexual topics” OR “Patient’s sexual health”	4	4	3	2

**Table 2:** Eligible articles from various database searches and search term combinations.

Search	Eligible Articles (Total 19) * = *Duplicates* (Total 10)
#1	1. Arikan, F., Meydanlioglu, A., Ozcan, K., and Canli Ozer, Z. (2015). 2. Baker, G. R. (2017). 3. Ek, G. F., Gawi, A., Nicolai, M. P. J., Krouwel, E. M., Den Oudsten, B. L., Den Ouden, M. E. M., … Elzevier, H. W. (2018). 4. Hoekstra, T., Lesman-Leegte, I., Couperus, M. F., Sanderman, R., and Jaarsma, T. (2012). 5. Li-Li Huang, Jing Pu, Li-Hua Liu, Xiao-Bo Du, Jin Wang, Jun-Ying Li, … Mei He. (2013). 6. Saunamäki N, Andersson M, and Engström M. (2010). 7. Saunamäki, N., and Engström, M. (2014). 8. Vermeer, W. M., Bakker, R. M., Stiggelbout, A. M., Creutzberg, C. L., Kenter, G. G., and Ter Kuile, M. M. (2015). 9. Yodchai, K., Hutchinson, A. M., and Oumtanee, A. (2018).
#2	1. Fitch, M. I., Beaudoin, G., and Johnson, B. (2013). 2. Maree, J., and Fitch, M. I. (2019). 3. Ussher, J. M., Perz, J., Gilbert, E., Wong, W. K. T., Mason, C., Hobbs, K., and Kirsten, L. (2013). 4. *Saunamäki N, Andersson M, and Engström M. (2010).
#3	1. Klaeson, K., Hovlin, L., Guvå, H., and Kjellsdotter, A. (2017). 2. Reese, J., Beach, M., Smith, K., Bantug, E., Casale, K., Porter, L., … Lepore, S. J. (2017). 3. Zeng, Y. C., Liu, X., and Loke, A. Y. (2012). 4. *Arikan, F., Meydanlioglu, A., Ozcan, K., and Canli Ozer, Z. (2015). 5. *Baker, G. R. (2017). 6. *Fitch, M. I., Beaudoin, G., and Johnson, B. (2013). 7. *Hoekstra, T., Lesman-Leegte, I., Couperus, M. F., Sanderman, R., and Jaarsma, T. (2012). 8. *Li-Li Huang, Jing Pu, Li-Hua Liu, Xiao-Bo Du, Jin Wang, Jun-Ying Li, … Mei He. (2013). 9. *Ussher, J. M., Perz, J., Gilbert, E., Wong, W. K. T., Mason, C., Hobbs, K., and Kirsten, L. (2013). 10. *Saunamäki N, Andersson M, and Engström M. (2010). 11. *Vermeer, W. M., Bakker, R. M., Stiggelbout, A. M., Creutzberg, C. L., Kenter, G. G., and Ter Kuile, M. M. (2015). 12. *Yodchai, K., Hutchinson, A. M., and Oumtanee, A. (2018).
#4	1. Martel, R., Crawford, R., and Riden, H. (2017). 2. Quinn, C., Platania, P. C., Bale, C., Happell, B., and Hughes, E. (2018).
#5	1. Akinci, A. (2011). 2. Evcili, F., and Demirel, G. (2018).

**Table 3:** Summary of the studies included in the review.

Author (Year)	Study Location	Aim	Study Design	Participants
Akinci (2011)	Turkey	To determine nurses’ comfort levels and factors affecting their comfort levels during clinical experiences, which include sexual topics.	Cross-sectional	141 nurses working at the medical and surgical units at two state hospitals in Hatay, Turkey.
Arikan et al. (2014)	Turkey	To determine the attitude and beliefs of nurses regarding sexuality and to establish the obstacles preventing them from offering counselling on sexuality.	Cross-sectional	162 nurses working in a University Hospital i.e., 88 from internal medicine, 58 from surgery, 5 from psychiatry, and 11 from obstetrics.
Baker-Green (2017)	UK	To explore nurses’ experiences of communicating with patients with an indwelling urinary catheter about sexual quality of life.	Qualitative semi-structured interviews	Nine registered nurses employed by the National Health Service and working in the district nursing service
Van Ek et al. (2018).	The Netherlands	To explore to which extent Dutch nurses working with patients receiving dialysis discuss sexual dysfunction and to identify possible barriers restraining nurses from discussing sexual dysfunction.	Cross-sectional	551 nurses
Evcili and Demirel (2018)	Turkey	To define the views of the nurses about the evaluation of the sexual health of the patients and the obstacles they experienced during the evaluation of sexual health.	Cross-sectional	188 nurses
Fitch, Beaudoin, and Johnson (2013)	Canada	To understand healthcare providers’ perspectives of the barriers to having conversations about sexuality in daily ambulatory cancer care and how these might be overcome.	In-depth semi-structured qualitative interview	34 cancer care professionals (nurses, physicians, social workers, and radiation therapists)
Hoekstra et al. (2012)	The Netherlands	To examine the current practice of discussing sexual health by heart failure (HF) nurses, and to explore which barriers prevent nurses from discussing sexuality.	Cross-sectional	146 nurses working with heart failure patients
Klaeson et al. (2017)	Sweden	To illuminate nurses’ experiences and opportunities to discuss sexual health with patients in primary healthcare.	Semi-structured qualitative interviews	9 primary healthcare nurses
Huan et al. (2013)	China	To investigate cancer department nurses’ attitudes and practices in response to pelvic radiation patients’ sexual issues in Sichuan, China.	Cross-sectional	128 nurses cancer care nurses
Martel, Crawford and Riden (2017)	New Zealand	To identify what facilitates primary healthcare nurses to discuss sexual health with youths.	Mixed methods	23 primary healthcare nurses
Maree and Fitch (2019)	Canada and Zimbabwe	To gain an increased understanding about the dialogue between cancer care professionals and cancer patients regarding the topic of sexuality.	Qualitative interviews in Canada and focus group discussions in Zimbabwe.	34 healthcare professionals in Canada and 27 Zimbabwean nurses engaged in a focus group discussion
Saunamäki, Andersson and Engström (2010)	Sweden	To describe registered nurses’ attitudes and beliefs toward discussing sexuality with patients.	Cross-sectional	88 registered nurses
Saunamaki and Engström (2014)	Sweden	To describe how RNs reflect on discussing sexuality with patients.	Qualitative interviews	10 registered nurses
Ussher et al. (2013)	Australia	To examine healthcare providers’ constructions of sexuality post-cancer, the subject positions adopted in relation to sexual communication, and the ways in which discourses and subject positions shape information provision and communication about sexuality.	Semi-structured qualitative interviews	38 healthcare providers (9 doctors, 11 nurses, 10 psychologists, and 8 social workers)
Quinn et al. (2018)	Australia and England	To gather information about how nurses working in mental health settings respond to sexual health issues within their routine practice: what sexual health issues nurses address during their consultations with mental health consumers; and their view on their role on promoting sexual health for mental health consumers.	Cross-sectional	303 nurses working in public mental health settings (Australia = 219; England = 84).
Zeng, Liu, and Loke (2012)	China	To describe Chinese nurses’ attitudes and beliefs with regard to discussing sexuality concerns with people with gynecological cancer, to investigate their current practice in addressing gynecological cancer patients’ sexuality concerns, and to explore the possible facilitators or barriers influencing these Chinese nurses’ practice.	Cross-sectional	202 nurses working in gynecological units
Yodchai, Hutchinson and Oumtanee (2018)	Thailand	To explore nephrology nurses’ perceptions of discussing sexual health issues with patients receiving dialysis.	Semi-structured qualitative interviews	20 nephrology nurses working in dialysis units
Reese et al. (2017)	USA	To characterize the experiences, needs, and intervention preferences of breast cancer survivors and healthcare providers with respect to patient–provider communication about sexual concerns in an effort to inform intervention development.	Qualitative interviews with HCPs 5 focus groups with partnered breast cancer survivors 4 interviews with unpartnered breast cancer survivors	28 women treated for breast cancer 11 healthcare providers (breast cancer oncologists and nurses)
Vermeer et al. (2015)	The Netherlands	To assess healthcare providers’ (HCPs) current psychosexual support practices, barriers to provide psychosexual support, and HCP needs for training and assistance.	In-depth qualitative interviews	30 HCPs involved in the care of women with gynecological malignancies

* HCPs = healthcare providers.

## Data Availability

Not applicable.
